# Cornelia de Lange Syndrome: From a Disease to a Broader Spectrum

**DOI:** 10.3390/genes12071075

**Published:** 2021-07-15

**Authors:** Angelo Selicorni, Milena Mariani, Antonella Lettieri, Valentina Massa

**Affiliations:** 1 Mariani Foundation Center for Fragile Child, Pediatric Unit ASST Lariana, 22100 Como, Italy; milena.mariani@asst-lariana.it; 2 Department of Health Sciences, Università degli Studi di Milano, 20142 Milano, Italy; antonella.lettieri@unimi.it (A.L.); valentina.massa@unimi.it (V.M.); 3 CRC Aldo Ravelli for Neurotechnology and Experimental Brain Therapeutics, Department of Health Sciences, Università degli Studi di Milano, 20142 Milano, Italy

**Keywords:** CdLSp, cohesins, WNT pathway, lithium

## Abstract

Cornelia de Lange syndrome (CdLS) is a genetic disease that exemplifies the evolution of knowledge in the field of rare genetic disorders. Originally described as a unique pattern of major and minor anomalies, over time this syndrome has been shown to be characterized by a significant variability of clinical expression. By increasing the number of patients described, knowledge of the natural history of the condition has been enriched with the demonstration of the relative frequency of various potential comorbidities. Since 2006, the discovery of CdLS’s molecular basis has shown an equally vast genetic heterogeneity linked to the presence of variants in genes encoding for the cohesin complex pathway. The most recent clinical-genetic data led to the classification of the “original syndrome” into a “clinical spectrum” that foresees the presence of classic patients, of non-classic forms, and of conditions that show a modest phenotypic overlapping with the original disease. Finally, the knowledge of the molecular basis of the disease has allowed the development of basic research projects that could lay the foundations for the development of possible innovative pharmacological treatments.

## 1. Introduction

Cornelia de Lange syndrome (CdLS) (OMIM #122470, #300590, #610759, #300882 and #614701) is a multisystem genetic disorder characterized by prenatal and postnatal growth retardation, microcephaly, distinctive facial feature, psychomotor retardation/intellectual disability, hirsutism, small hands and feet or limb malformations. The incidence of the syndrome has been estimated within 1:10,000 to 1:30,000 live births [1].

The natural history of the disease includes multiple chronic medical problems, pivotal for properly planning and addressing the follow-up of the affected patients. Several behavioral comorbidities have also been described. The first description of the disease was by a Dutch pediatrician, Cornelia de Lange, who observed it in two children in 1933 [2]. Two letters to the editor by and Meinecke and Hayek (1990) [3] reported that a similar clinical association had previously been described by Brachmann and Vrolik in 1916 and 1849, respectively [4,5]. For this reason, the syndrome has also been named Brachmann de Lange.

Aside from the original description, over time several authors have tried to define a list of diagnostic criteria, starting from Berg et al. [6], to Preus et al. [7] and Kline et al. [8]. With the increase in the number of observed patients, the awareness of the existence of a wider variability of clinical expression than initially hypothesized grew. Accordingly, Van Allen et al. [9] suggested the existence of three different clinical subtypes: a classic one (type I), a mild one (type II) characterized by mild to borderline psychomotor retardation, less severe pre- and postnatal growth deficiency, and the absence of (or less severe) major malformations, and a type III form (“phenocopies”) including patients who have phenotypic manifestations of CdLS which are caused by chromosomal aneuploidies or teratogenic events. The observation of mildly affected CdLS patients was confirmed by other research groups in the same year [10,11,12,13]. In the absence of a confirmatory genetic test, the great variability of clinical expression led in the past many groups to diagnose CdLS in multiple patients on a clinical basis only. With the subsequent progressive discovery of the complex and heterogeneous biological basis of the disease, these patients were included in the molecular confirmation analysis, conditioning in some way the detection rate of the same genetic tests.

In 2018, a large international group of professionals, scientists and two patient- group representatives published an in-depth consensus statement [14] in which the clinical variability and the genetic heterogeneity of the disease were classified as a spectrum (CdLSp). The authors defined a detailed diagnostic algorithm based on the combination of “cardinal” and “suggestive” features, assigning each a specific score. According to the final score reached by the patient, he/she can be classified as classic or non-classic CdLS. Table 1 describes this algorithm. It is important to underline that the definition of classic and non-classic CdLS do not overlap with the old definitions of classical and mild type. Moreover, it was stated that all patients belonging to the CdLSp show anomalies of the various cohesin complex genes, but not all patients with anomalies of these genes can be classified as CdLSp. In other words, CdLSp belongs to a broader group of conditions, named cohesinopathies, which includes patients carrying variants in one of the different genes belonging to the cohesin complex, and whose phenotype might or might not be related to CdLS.

In recent years, computer-assisted image analysis systems have become increasingly helpful for professionals and clinical diagnosis [15]. For example, Face2Gene (https://www.face2gene.com/ accessed on 10 September 2019) is of support for facial phenotype identification in rare genetic disorders. In 2020, Latorre-Pellicer et al. reported their experience in evaluating Face2Gene use and sensitivity in the clinical diagnosis of 49 molecularly confirmed CdLS patients [16]. CdLS diagnosis was included in the top five possibilities in 97.9% of cases, and it was the first suggested diagnosis in 83.7% of cases. These impressive results are probably due to the high percentage of classic CdLS patients according to the International Consensus statement which made up the cohort. Further analysis of a more heterogeneous group of patients will provide even more accurate data on the effectiveness of this system.

## 2. Biological Basis of CdLS

**Figure 1:**
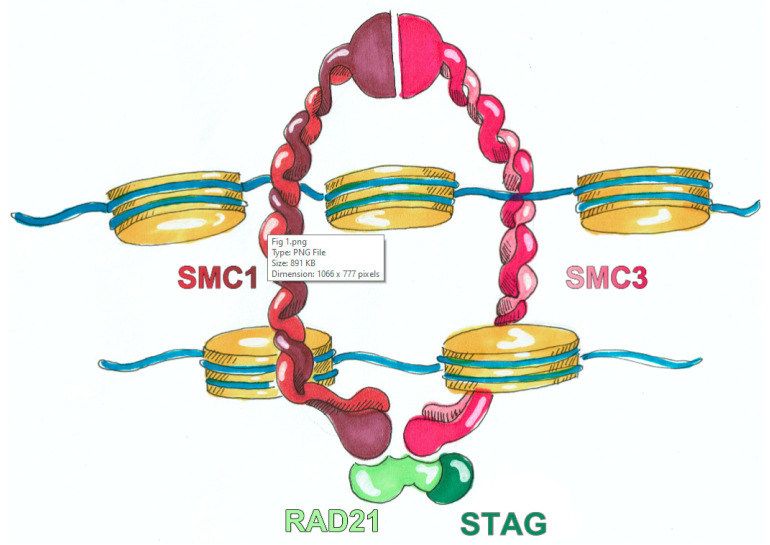
The ring-shaped cohesin complex is formed by a multi-subunit core: structural maintenance of chromosomes (SMC3 and SMC1A), RAD cohesin complex component (RAD21), and cohesin subunit SA (STAG). Cohesin complex can entrap the sister chromatids and regulates the separation of sister chromatids; furthermore, can modulate the chromatin conformation and affects normal gene expression.

To date, six genes of the cohesin complex have been identified and associated with CdLS: nipped-B like protein (*NIPBL*), structural maintenance of chromosomes 1A (*SMC1A*) and 3 (*SMC3*), double strand break repair protein rad21 homolog (*RAD21*), bromodomain-containing protein 4 (*BRD4*), histone deacetylase 8 (*HDAC8*) [17]. Most of the affected individuals show de novo pathogenic variants in one of these genes, with the most prevalent variant in *NIPBL* (60–70%) [14]. The proteins encoded by CdLS causative genes have been identified as structural or regulatory components of the cohesin complex. The core of cohesin complex (Figure 1) is a multimeric system composed by different subunits SMC1A, SMC3, RAD21 and STAG, it is evolutionarily conserved and exerts its canonical role in sister chromatid cohesion, and non-canonical role in gene expression regulation. The other misregulated proteins, that are not comprised in the core, are cohesin- or chromatin-associated factors genetically different from cohesin but whose variants were found in patients presenting features of CdLSp phenotype through multiple next-generation sequencing (NGS) technologies [18]. The ring-shaped cohesin complex was initially identified for its key role in chromosomal integrity maintenance during the cell cycle phases but now it is known that the interplay with the chromosome loader NIPBL and the sequence-specific DNA binding protein CTCF (CCCTC-binding factor insulator protein) organizes the genome into TADs (topologically associated domains), chromatin loops, and contact domains [17]. Thus, the 3D DNA organization driven by cohesin complex and its associated regulatory proteins helps the contact of sequence elements, including enhancers and promoters, finally orchestrating gene expression [19]. In accordance with this fundamental regulatory role, CdLS cell lines do not display abnormalities in sister chromatid cohesion [20,21] but rather in genes and proteins expression and production [22,23,24,25,26], suggesting that CdLS etiopathology is due to altered transcriptional regulation derived from an impaired function of the cohesin complex in 3D chromatin organization. Furthermore, many genes have also been found weakly transcriptionally dysregulated in CdLS animal models [27]. 

Besides sister chromatid cohesion and gene expression regulation, a role has recently been proposed for the cohesin complex in DNA damage signaling and repair [28], raising the possibility of novel molecular mechanisms for CdLS. Specifically, Singh et al. demonstrated that insufficient cohesin levels in *Nipbl* and *Hdac8* heterozygous mouse models led to persistent DNA damage in placental cells, resulting in increased senescence and cytokine secretion and ultimately in diminished embryo health and viability [29]. Furthermore, Olley and colleagues showed that CdLS-associated variant in *BRD4* delays the cell cycle by perturbing regulation of DNA repair and increasing DNA damage signaling. Similarly, *NIPBL* deficient cells derived from CdLS patients exhibited an increase in DNA damage response, suggesting the importance of this signaling in the etiology of CdLS [30]. In addition, the RNA-sequencing of patient specific *NIPBL+/−* iPSC lines identified an altered expression of many mRNAs, pseudogenes, and non-coding RNAs leading to upregulation of gene sets with functions in epigenetic control and downregulation of WNT pathway [31].

WNT signaling alterations have been associated with a plethora of CNS abnormalities and canonical WNT pathway was found to be perturbed in CdLS models [32,33,34,35]. In the contest of embryonic development, the WNT signaling plays a fundamental role in all steps of development by regulating cell proliferation, differentiation, migration, genetic stability and apoptosis, as well as by maintaining adult stem cells and pluripotent state [36]. The many malformations characterizing the CdLSp phenotype have been hypothesized to be derived from developmental WNT/β catenin pathways deregulation. 

## 3. Clinical Features 

**Figure 2:**
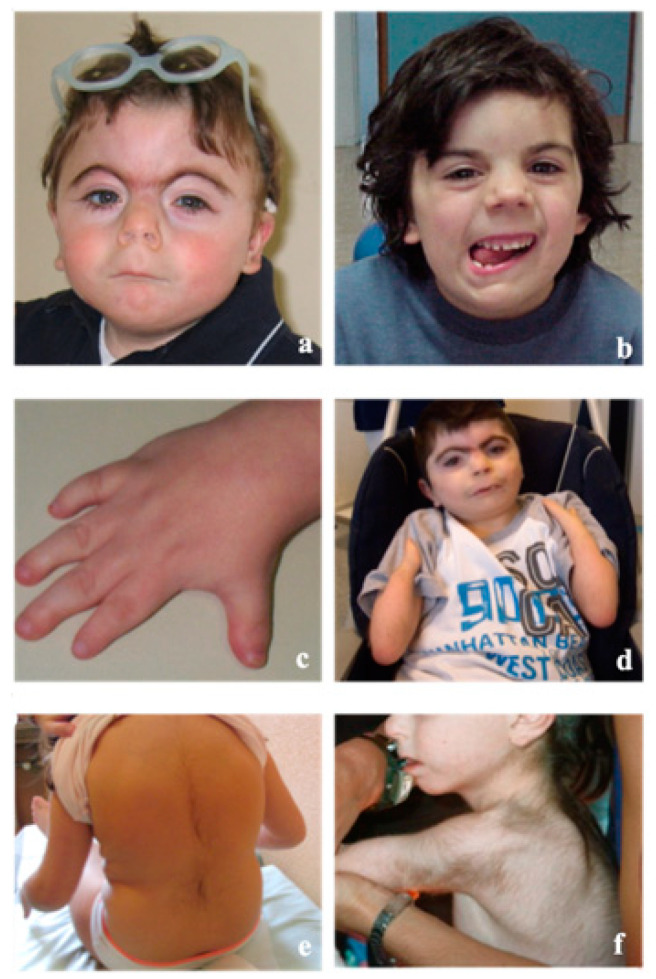
Main clinical features in CdLSp. Facial phenotypes: classic phenotype (**a**) and mild phenotype (**b**). Typical small hand with brachydactyly, proximal thumb and clinodactyly of the fifth finger (**c**) or limb reduction defect (**d**). Typical hirsutism of the back localized (**e**) and diffuse (**f**).

Patients affected with classic CdLS show a quite unique clinical “gestalt” (Figure 2) which can be recognized in the neonatal period. They can present a short neck with low posterior hairline and hirsute forehead. The eyebrows are with synophrys and eyelashes are thick and long; palpebral ptosis is quite frequent with different level of severity. Ears are lowset, midface can be flattened, the nose is short and the philtrum long and featureless. The mouth has a thin upper lip with down-turned corners; a high palate, widely spaced teeth and micrognathia can be observed. Typically, hands and feet are small with shortening of the first metacarpal, proximally placed thumb, brachydactyly, clinodactyly of the fifth finger and single palmar creases. One third of patients can show reduction defects of the upper limbs of variable severity (from oligodactyly to ulnar deficiency to absent forearm). Some degree of reduction in elbow mobility is quite frequent only rarely due to a real radioulnar synostosis. Hirsutism, particularly of the face, lower back and forearms, cutis marmorata and small nipples are observed in the majority of patients.

Patients with CdLSp classically show a prenatal and postnatal growth delay; specific growth charts have been published to properly clinically follow these patients [37]. Such charts were designed before the discovery of the genetic basis of the disease, and hence they should be updated on the basis of genotypic differences. Notably, data from teenagers and young adults underline that CdLSp patients tend with time to develop overweight and/or outright obesity in a fair percentage of cases.

Major malformations have been observed in various organs and systems. Table 2 describes the different percentage of involvement. Apart from upper limb anomalies, none of these malformations is crucial from a diagnostic point of view. It is important to keep in mind that presence of intestinal malrotation, reported in between 5% and 10% of patients, is related to the risk of acute cecal volvulus, which represents a real surgical emergency and one of the most frequent causes of mortality in patients. 

Psychomotor and intellectual development are classically delayed, showing a variable degree of severity, from mild to profound, with the majority of patients showing moderate to severe retardation. Communication skills are particularly impaired with expressive language that appears to be more compromised than the receptive one [38]. The behavioral phenotype of CdLSp patients has been deeply analyzed. It is well known that anxiety, mood disorders, impulsivity, challenging and self-injurious behavior, repetitive behavior are present in many patients. Self-injurious behavior, evident in about 56% of patients, seems to be related to severity of intellectual impairment, impulsivity, more compromised communication and *NIPBL* variant. Pain related to various medical comorbidities should always be considered as a possible reason for the onset of a self-injurious behavior.

As previously stated, natural history of CdLSp patients is characterized by the presence of many possible medical comorbidities that should be considered by pediatricians in the clinical follow-up. Their detailed description is beyond the scope of this review, so they are listed in Table 3.

Quite recently, data on nutritional issues in CdLSp patients have been published, showing that their diets are frequently unbalanced (76%) both from a quantitative and qualitative point of view. However, the authors did not find a direct correlation between patients’ BMI and the nutritional imbalance, hypothesizing an increased metabolic rate for explaining the growth pattern [39]. Similarly, Matute-Llorente et al. performed a detailed research on bone health and body composition in a small number of *NIPBL* CdLS patients presenting a normal adiposity but a reduced lean mass if quantified by DEXA [40]. Both these papers underline the need for further studies for elucidating the metabolic basis of the growth pattern of CdLSp patients.

Definition of an assessment procedure for the severity of single patient has been attempted by several groups. Kline et al. [14], Selicorni et al. [41], and Bhuiyan et al. [42] suggested specific severity scores based on the combination of different clinical features that classified CdLSp patients in mild/moderate/severe [14,41] or in classical/mild and possible [42]. Moreover, Cereda et al. [43] generated a prognostic score with the aim of early predicting the degree of severity of intellectual disability of a single patient based on a combination of clinical and genetic elements that can be easily defined in the first months of life. Currently none of these scores is considered sufficiently satisfactory on the base of the expectations of the parents.

**Table 3:** Prevalence of medical problems in CdLS patients [14].

Medical Problem	Prevalence
Gastrointestinal problems	
-Feeding problems	frequent
-Enteral nutrition	40%
-Gastro esophageal reflux	60–75%
-Batter esophagus	9%
-Eosinophilic esophagitis	16%
-Constipation	10–15%
-Gassiness	48%
Neurology	
-Seizures	45% (SMC1A gene) 15% (NIPBL gene)
-Autonomic nervous system disfunction	81% (mild) 26% (severe)
-Sleep problems	12–72%
Orthopedics	
-Perthes disease	4%
-Leg length differences	46%
-Congenital hip dislocation	10%
-Scoliosis	One third of patients after 10 years
-Kyphosis	A quarter of patients
-Joint contractures	18–25%
-Bunions	75%
Visual problems	
-Palpebral ptosis	37% (unilateral) 44% (bilateral)
-Blepharitis	25%
-Nystagmus	14–17%
-Strabismus	16–26%
-Visual impairment	44–53%
-Pigmented peripapillary ring	83%
Hearing problems	
-Conductive hearing loss	75%
-Neurosensorial hearing loss	25%
-Otitis media with effusion	80–85%
Immunological defects	33% (only one specific report) [44]
Thrombocytopenia	rare
Cancer	No increase of risk

## 4. Genotype–Phenotype Correlations

From the beginning of the genomic history of CdLS, the aim of researchers has been that of finding possible correlations between clinical phenotype and molecular data as soon as new genes have been discovered. Currently, despite the growing amount of information, major limits in this effort are represented by the enormous difference, in numerical terms, between patients with variants in the major gene (*NIPBL*) and patients carrying variants in the other genes of the cohesin complex. In fact, it is well known that the latter represent a minority (5–10%) among those who are now classified as CdLSp subjects [14].

*NIPBL* gene: about 70% of CdLS patients show variants in this gene. It is responsible for the great majority of the more classical and more severely affected cases. This is particularly true for those subjects with the loss of function variants (microdeletions, exon deletions, truncating, non-sense, splicing and frameshift). These patients are the only ones with classical limb reduction defects. Patients with missense variants usually show a better prognosis from both clinical and developmental issues [41,42,45]. Parenti et al. recently described a patient with a severe phenotype and typical CdLS facial dysmorphism carrying a pathogenic variant in *MAU2*, interactor of NIPBL. This variant appears to affect *NIPBL* coding sequence and therefore causing *NIPBL* haploinsufficiency [46]. 

*SMC1A* gene: variants in this gene represent about 5% of genetically determined subjects [47]. *SMC1A* patients show some differences from a dysmorphic point of view compared with *NIPBL* ones. Their auxological data at birth are frequently normal. Motor development milestones are achieved earlier than in *NIPBL* patients and their intellectual performance is generally higher [48]. However, about 40% of patients with variants in *SMC1A* show a completely different phenotype, characterized by a severe epileptic encephalopathy with Rett-like features [49,50]. 

*SMC3* gene: this represents a very rare cause of CdLS. Patients bearing variants in this gene show an atypical CdLS phenotype characterized by short stature, intellectual disability, and quite high prevalence of heart malformation (50–60%) [51].

*RAD21* gene: variants in *RAD21* gene are rarely observed as a cause of CdLS, and the clinical phenotype is considered atypical [52,53]. Krab et al. reported clinical and molecular data of a wide cohort of *RAD21* patients confirming that they show a quite attenuate CdLSp phenotype particularly concerning facial morphology, limb anomalies, behavior and cognitive development [54].

*BRD4* gene: the reported number of patients showing haploinsufficiency in this gene so far is too low to describe a specific phenotype [55].

*HDAC8* gene: patients with variants in this gene represent about 5% of the global CdLS cohort. These subjects can have both a classical and an atypical phenotype [24,56]. Male patients are more severely affected as the gene locus is on the X chromosome. At a clinical level, these patients quite frequently show hypertelorism, wide anterior fontanel and a happy personality. The severity of the cognitive involvement is generally worse than that *SMC1A*, *SMC3*, *BDR4* and *RAD21* patients [57].

Last, the *ANKRD11* gene should be counted, as it was firstly associated with KBG syndrome and then associated with CdLS. It is known that the phenotypic overlapping between these two syndromes can sometimes be very strong [58]. Parenti et al. reported clinical and molecular data of 23 patients with *ANKRD11* variants, showing that all but two of them received an initial clinical diagnosis of either CdLS or KBG syndrome; in only five patients did the phenotype at later stages of child development allow a final diagnosis of KBG syndrome [59]. 

In 2010, Castronovo et al. described a CdLS patient presenting a somatic mosaicism for a *NIPBL* truncating variant discussing whether mosaicism could be the reason for the less severe phenotype of the child [60]. Later (2013), Huisman et al. described a high prevalence of somatic mosaicism (23%) in their cohort of CdLS patients, suggesting that somatic mosaicism could be more frequent than expected in CdLS. Interestingly, signs and symptoms did not differ between patients with mosaicism and those with germline variants [61]. The real prevalence and the phenotypic consequences of somatic mosaicism in CdLS is still unclear and it needs further study. 

A CdLS-like phenotype have been observed also in patients harboring variants in other genes associated with CdLSp overlapping phenotypes. Table 4 summarizes such information.

## 5. From Biological Basis to Future Treatment Options

To date, no definitive cure exists for CdLSp patients; only specific and localized symptomatic treatments, sometimes alongside surgery. CdLSp patients are characterized by intellectual disabilities, which range from profound to mild, and behavior of the autism-spectrum disorders indicating neural development alterations [14]. The disruption of gene regulation during critical steps of early embryonic development contributes to a WNT signaling alterations that has been associated with a plethora of CNS abnormalities. To date, different studies have shown that the WNT canonical pathway is altered in CdLS models [32,33,34,35], suggesting it and its downstream effectors as a treatment target option. 

### 5.1. Lithium

Lithium is a well-known drug and an effective therapeutic agent for bipolar disorders [62], recently implemented for amyotrophic lateral sclerosis treatment as well, and acts as a specific inhibitor of the conserved WNT transducer GSK3 [63]. Notably, it has been shown that lithium treatment, by modulating the WNT pathway, was able to rescue phenotypes associated with CdLS in *nipblb*-loss-of-function zebrafish embryos [32] as well as in *Drosophila nipbl* model and murine and human in vitro models [35]. These data suggest that lithium might be used to at least partially ameliorate CdLS phenotype also in CdLS human fetuses, by maternal administration, since it is frequently prescribed to pregnant women for the treatment of bipolar disorder [64]. Yet, the use of lithium during pregnancy raises concerns, mainly with respect to the negative effects on embryo development. Two independent groups have shown the consequences of in utero lithium exposure on embryos by meta-analysis studies. Lithium administration in pregnant women during the first trimester increased the risk of fetus malformations and neonatal readmission within one month postpartum [65]. On the other hand, while most of preclinical studies reported adverse effects of prenatal exposure to lithium on neurodevelopment or behavior, clinical studies regarding intra uterine lithium exposure do not show abnormal fetus development [66]. Alternatively, a possible use of lithium in the treatment of CdLS patients might be considered in the first years of life, when newborn brain is still undergoing developmental and plasticity processes [67], with the aim of ameliorating cognitive and behavioral features. Lithium has also been proposed for a clinical trial in the context of fragile X syndrome for the treatment of behavioral aspects [68]. A selective inhibitor of GSK3α has been recently developed to avoid the toxic effects derived lithium-dependent inhibition of both GSK3 paralogs, and it was shown to successfully correct some phenotypes features of fragile X syndrome mouse models [69].

### 5.2. L-Leucin

Besides lithium treatment, L-leucine administration has been proposed. The reduced translation has been suggested to be a common feature in cohesinopathies animal models. L-leucine treatment during zebrafish development has been shown to rescue translation through mTOR pathway stimulation. Specifically, Xu and colleagues showed that in zebrafish embryos, the downregulation of cohesin complex genes by morpholino injection, resulted in multiple developmental defects typical of the CdLS phenotype, which were partially corrected by treatment with L-leucin. These data suggest that translation could be an interesting therapeutic target and that CdLS patients could benefit from this [70].

### 5.3. Antioxidant

At a cellular level, CdLS presents cell growth arrest, apoptosis, genome instability and premature aging, hallmarks of high levels of oxidative stress [71]. Thus, the reduction of reactive radical oxygen molecules presents a potential therapeutic target, and antioxidants have been suggested for drug repurposing with a clinical potential in improving CdLS phenotype. Treatment with ascorbic acid and riboceine drugs led to the extension of in vitro lifespan of CdLS cell lines and zebrafish model by reducing both the level of oxidative stress and genome instability [26]. In addition, it has been shown that N-acetyl-cysteine protects the DNA from damage and prevent cell death, in cerebellar cells [72,73]. A randomized clinical trial for the use of N-acetyl-cysteine in the treatment of neurodevelopmental disorders in CdLSp patients is estimated to start within this year (https://clinicaltrials.gov/ct2/show/NCT04381, accessed on 10 September 2019). 

## 6. Conclusions

CdLS could be considered to be an archetype of the successful interlink between bench and bedside research. Indeed, the two lanes have developed in parallel while bridging the gaps in knowledge over the decades and, as a result of this shared effort, CdLS is now a well-defined rare genetic disorder. Furthermore, thanks to the advancement in understanding of CdLS, the information gained has led to the hypothesizing of future therapeutic approaches, while at the same time sustaining the improvement of daily care and planned follow-up.

## Figures and Tables

**Table 1:** Diagnostic algorithm as suggested by the Consensus Statement.

Cardinal Features	Score
Synophrys (HP:0000664) and/or thick eyebrows (HP:0000574)	2
Short nose (HP:0003196), concave nasal ridge (HP:0011120) and/or upturned nasal tip (HP:0000463)	2
Long (HP:0000343) and/or smooth philtrum (HP:0000319)	2
Thin upper lip vermilion (HP:0000219) and/or downturned corners of mouth (HP:0002714)	2
Hand oligodactyly (HP:0001180) and/or adactyly (HP:0009776)	2
Congenital diaphragmatic hernia (HP:0000776)	2
**Suggestive Features**	
Global developmental delay (HP:0001263) and/or intellectual disability (HP:0001249)	1
Prenatal growth retardation (<2 SD) (HP:0001511)	1
Postnatal growth retardation (<2 SD) (HP:0008897)	1
Microcephaly (prenatally and/or postnatally) (HP:0000252)	1
Small hands (HP:0200055) and/or feet (HP:0001773)	1
Short fifth finger (HP:0009237)	1
Hirsutism (HP:0001007)	1
**Interpretation of the Score**	
Score ≥11 points, of which at least 3 are cardinal features	classic CdLS
Score between 9 or 10 points, of which at least 2 are cardinal features	non-classic CdLS
Score between 4–8 points, of which at least 1 is cardinal feature	molecular testing for CdLS indicated
Score <4 points	insufficient to indicate molecular testing for CdLS

**Table 2:** Major malformations described in CdLS [14].

Malformation	Prevalence
Heart malformations (no specific defect)	25%
Palate	20%
Eyes Unilateral or bilateral nasolacrimal duct obstruction	60–80%
Central Nervous System	47% (in the wider cohort reported) [35]
Limb defects	About one third
Urinary tract	10%
Genitalia	
Cryptorchidism	80%
Micropenis	37%
Hypospadias	9%
Bicornuate uterus	19%
Gastrointestinal system	
Intestinal malrotation	5–10%
Pyloric stenosis	7%
Diaphragmatic Hernia	rare

**Table 4:** CdLS-like phenotypes.

Gene	Overlapping Syndrome
*ANKRD11*	KBG syndrome
*AFF4*	CHOP syndrome
*EP300*	Rubinstein-Taybi syndrome
*KMT2A*	Wiedemann-Steiner syndrome
*TAF6*	Alazami-Yuan syndrome
*SETD5*	Mental retardation, autosomal dominant 23
*ARID1B*	Coffin-Siris syndrome
